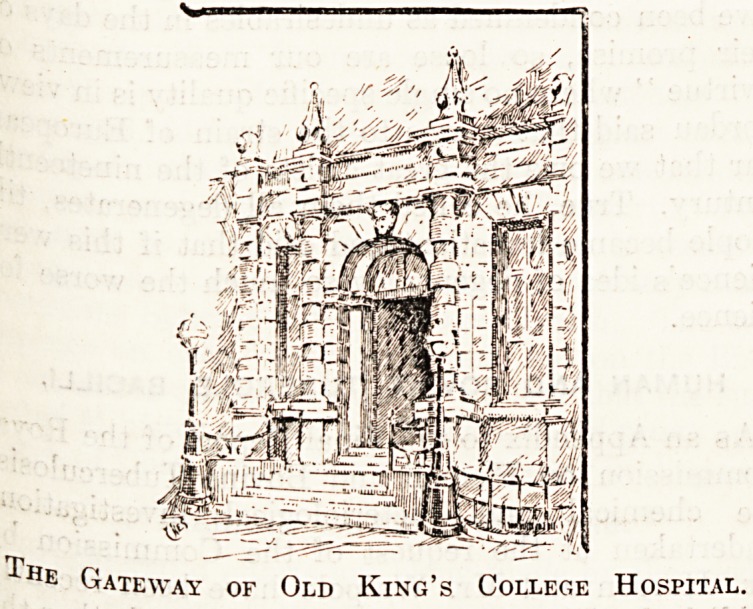# Hospital and Institutional News

**Published:** 1913-07-19

**Authors:** 


### HOSPITAL AND INSTITUTIONAL NEWS.

### THE KING AND ARMY HOSPITALS.

The annual Church Parade of the Liverpool Territorials on Sunday last was one of the most impressive features of the Royal tour in the North. The King and Queen were present at St. George's Hall, Liverpool, and after the service many interesting presentations, both institutional and medical, took place: Sir James Barr, president of the British Medical Association; Dr. Caton, late honorary colonel of the Royal Army Medical Corps of the West Lancashire Division; Miss Glover, principal matron of the 1st Western General Hospital connected with the Division; and Colonel Jackson, commanding the Western Hospital Section, were among them. The King also inspected the fifty sisters and nurses of the 1st Western General Hospital. These presentations were the most intimate feature of the parade, and testify to the importance which the King attaches to the hospital side of the work.

### THE LAST OF OLD "KING'S."

On Monday night the old King's College Hospital closed its institutional life in Portugal Street, and in consequence there was held a reunion of past and present students to commemorate the past by a last supper. In the early part of the evening the Matron, Miss Rae, received a bouquet. The health of "Mary" was drunk. Like her prototype, Red Lion Mary of pre-Raphaelite fame, the Mary of this toast has a fine record of service, having been doctors' housemaid for twenty-three years. The supper was followed by a concert, and Professor Urban Pritchard gave the toast of the "Old Hospital and the New," and enlarged, as who would not with such a text before

The Gateway of Old King's College Hospital.

him, on the changes in surgery and medicine which had occurred since he first became connected with the institution half a century ago. He made it plain by what paradox of progress the old building had become insufficient for its needs by remarking that King's College had been the first hospital to start special departments, and that these had been the doom of the old building through the constant demand for more beds which they occasioned. The evening was an interesting landmark, and Mr. W. H. Smith, the chairman of the hospital, and Sir Watson Cheyne also spoke of past achievements and the hopes of the future. The next date to record will be in October, when the institution will re-open on Denmark Hill, where six hundred beds will take the place of the two hundred and fifty which completed their period of service on Monday night.

### THE NEW HOSPITAL IN JUDD STREET.

The new buildings of the Central London Ophthalmic Hospital in Judd Street, St. Pancras, were opened by the Duchess of Albany on Tuesday afternoon. In an interesting speech Mr. T. B. Archer, chairman of the committee of management, recalled that it was just two years ago since the Duchess of Albany had laid the foundation-stone. The new buildings contain two operating theatres, one for in- and one for out-patients; a bacteriological and pathological department, *x*-ray room, library and museum, and extensive rooms for out-patients. Twenty-four beds are provided for in-patients, a number which it is hoped to increase to forty. A long, uphill fight has raised the £20,000 which has been spent, but funds are asked for to complete the equipment. To appreciate the new buildings it is necessary, of course, to study the plans and arrangement in detail, which we hope to give our readers an opportunity of doing next week.

### THE COMMISSIONERS AND MENTAL RESEARCH.

The reader who takes up the latest Report of the Commissioners in Lunacy, which was issued as a Blue-book last Saturday, will anxiously turn to see what deductions follow from the mass of figures showing the average annual increase of 2 per cent. which has marked the last ten years. The control of mental disease, the Commissioners remark, lies rather at present on its preventive than its therapeutic side. In other words, a fuller knowledge of its causation must be gained, and this means the prosecution of scientific research. If any of the recently discussed schemes are to be realised, this opens up a new instance of usefulness to the mental hospital. As Dr. Harding, the medical superintendent of Berry Wood, Northampton, pointed out in a recent interview in our columns: "There are existing centres in our medical schools around which local authorities could be grouped. At present it is illegal for such authorities to contribute towards the upkeep of such laboratories. This should be permitted and on the committee which is authorised to spend such funds there should be representatives of the bodies who contribute to them." It is because it is impossible for the ordinary individual mental hospital to undertake such work that the principle of Government grants in aid of research is to be welcomed. When research is put upon a national footing a way will be found for utilising the stores of material now untouched in the mental hospitals.

### TUBERCULOSIS ACTIVITY IN BIRMINGHAM.

The Birmingham scheme for the treatment of tuberculosis includes, in addition to the tuberculosis centre, described on page 466, three municipal sanatoria, while another, which is supported by the Hospital Saturday Fund, is available for patients from the city. The chief sanatorium officer is also tuberculosis officer, and the tuberculosis centre is worked by the staff of the largest sanatorium, which is within the city boundary. It is found that this arrangement results in increased efficiency. Public bodies contemplating arrangements for the treatment of tuberculosis cannot do better than inspect the Birmingham scheme.

### BIRMINGHAM AND PUERPERAL SEPSIS.

A special ward for puerperal fever cases was opened last week at the Birmingham and Midland Hospital for Women, Sparkhill. The new ward, which is a self-contained unit, provides accommodation for twenty-five patients, and possesses on its south side a sheltered verandah with six beds for open-air treatment. The ward has been built at a cost of £6,000, and is the latest development of a scheme embarked upon two years ago, when the hospital came to an arrangement with the City Council to reserve two beds for septic cases. Later this number had to be increased to seven, and now the Health Committee, while taking no share in capital expenditure, is to contribute three-fifths of the annual cost of maintenance. Puerperal fever is a disease, or should we say symptom, which has striking reactions. As Dr. Robertson, the Medical Officer of Health, reminded his audience at the opening, there has been a constant difficulty in getting a medical man to attend cases that arose in houses where there was only a nurse or a midwife. He could understand this, he added, for such attendance always carried with it a stigma, and tended to interfere with ordinary work. The institution of the new ward, he hoped, would do away with these difficulties. Again, take the question of the notification of puerperal fever, which is enforced in Birmingham. Readers of our article in The Hospital of July 5 will want no reminding of how misleading are statistics dealing with the incidence of puerperal fever from the temptation of practitioners to record none save the most patent cases. If the new ward relieves medical men of a possibly unmerited stigma, by receiving suspected cases at the earliest date, it may do more to aid perfect Listerian technique than notification will ever do.

### DOLES FOR IN-PATIENTS.

A rather curious bequest has been included as a suggestion in the will of the late Colonel Sir Charles Stoddart, V.D., of Rotherham, managing director of an iron and steel company. He left £8,000 for charitable objects generally and bequeathed the residue of his property for a fund to be applied in perpetuating his memory in the borough, and suggested two alternative ways in which this might be done. Almshouses was the first idea that apparently commended itself to the testator, but his second thought is for a fund to provide doles for poor people who have been under treatment as in-patients in the Rotherham Hospital. It does not appear whether the fact of having been a poor in-patient at this institution is sufficient qualification for a pension, or if, as is perhaps more probable, the fund which he suggests should act as a sort of supplementary Samaritan fund, to assist patients on the road both to true convalescence and to a livelihood. The idea, however, is an interesting tribute to the importance attaching to the patient at the moment of leaving the institution, and should it be acted upon it will be interesting to hear how the Rotherham Hospital authorities propose to carry out the bequest.

### WAR AND EUGENICS.

Is eugenics to be used as a stalking-horse for the introduction of compulsory military service? We ask the question not to ridicule the new science, but because it arises out of a lecture on the "Eugenics of War" which Professor Starr Jordan, Chancellor of Stanford University, delivered early this week. His contention was that wars spoil a nation's breed, because the physically unfit are left behind to propagate while the fit are absent fighting. If this could be proved, which seems quite open to doubt, the most convenient way of keeping the stock "pure" would be, of course, to enrol all the men in the ranks. But the consideration that makes us hold this contention lightly is the assumption on which it rests, that the men who are fit for war are the men whom it is most desirable to propagate. The whole difficulty of eugenics lies in the fact that any alternative to our present promiscuity has to face the complex question: What sort of person do you want? The only true answer at present is that we do not know, while it seems very possible that those whom the world has decided to call great would have been condemned as undesirables in the days of their promise, so loose are our measurements of "virtue" where no single specific quality is in view. Nordau said that it was to the strain of European war that we owe the great artists of the nineteenth century. True, he called them all degenerates, till people became impatient and said that if this were science's idea of degeneracy so much the worse for science.

### HUMAN AND BOVINE TUBERCLE BACILLI.

As an Appendix to the Final Report of the Royal Commission on Human and Bovine Tuberculosis, the chemical and bacteriological investigations undertaken at the request of the Commission by Dr. Harden and Mr. Walpole have been recently published. The object was to ascertain whether the cultural and chemical differences between the two types were sufficiently marked to differentiate one from the other. The general tenour of the report is that no such definite physiological differences exist, and certain alleged differences are shown to be capable of quite natural explanation on other grounds, such as the times of incubation, the weight of the organisms, and so forth. Considering how precise the cultural methods of modern bacteriology have become in the separation of different varieties of bacteria, the findings of Dr. Harden's report certainly support the views of those who deny that human and bovine tubercle bacilli are to be separated by any hard-and-fast line.

### THE LATE DR. McCASKIE.

The death of Dr. Norman McCaskie at Southport removes a member of the medical profession exceedingly well known, until his retirement, in the West End of London. Dr. McCaskie came to London from Scotland many years ago, and speedily built up a very large practice in South Kensington. Blessed with a rich Scottish accent, which he was never at any pains to get rid of, his personality was equally well marked; and few successful medical men can have made fewer enemies than he did. Both his sons eventually joined him in practice, and some two or three years ago he relinquished his patients entirely to them, retiring to Southport, where one of his daughters is the wife of a medical man. Dr. McCaskie was sixty-four years of age, and the news of his death will be a shock to many of those who knew him in London. We understand that melanotic sarcoma was the cause of his decease.

### A MINISTER OF NATIONAL HEALTH.

The proposal that there should be a Minister of Health with a seat in the Cabinet is no new one, but its advocates have generally been those in favour of a regular State medical service. It has been upheld as the crowning symbol of a great change. At a conference of insurance representatives at Birmingham last week, however, a National Health Minister was advocated on the ground that a great change had taken place already, and that a new department was in actual existence, and only awaiting a Minister with Cabinet rank to symbolise its achievements up to date. This department is, of course, the State Insurance Office, as revealed in the first report of the Insurance Commissioners. It was urged, further, that as one of the great spending departments it should be accorded independent existence and separated from the Treasury. This last may be a good suggestion, but it seems a thoughtless misnomer to confuse a compulsory State Insurance Scheme with National Health. The two impinge on each other no doubt, but on the lines of the above-quoted discussion we fear that the suggested Minister of National Health would prove only a sort of Chief Insurance Commissioner in disguise.

### ITINERANT BUTTER VENDORS.

Interesting efforts are being made by the medical and sanitary staff at Wandsworth to deal with itinerant butter vendors, who, although residing outside, yet trade in the borough. Dr. P. Caldwell Smith, M.A., M.D., D.P.H., Medical Officer of Health, states that the article for sale proves on analysis to be margarine with only a very small percentage of butter. The itinerant vendors solicit orders and dispose of their goods by means of a pony and cart, and when they find that the so-called "butter" is giving satisfaction to customers, they ask for recommendations to their friends. One lady who assisted an inspector to capture one of these vendors told him that she had recommended the man to no fewer than six of her friends, and that she had been recommended to him herself. Dr. Caldwell Smith is of opinion that by a system of registration these fraudulent traders could be kept in check, but at present no system exists.

### DEATH OF PROFESSOR GOTCH.

Professor Gotch, F.R.S., Waynflete Professor of Physiology at Oxford, has died at the age of sixty. His varied educational studies at University College, London, found expression and acknowledgment in the degrees in Arts and Natural Science which he obtained before qualifying M.R.C.S. in 1881. He became Sharpey Scholar in Physiology at University College, London, and acted as demonstrator to Burdon Sanderson when the latter was appointed first Waynflete Professor of Physiology on the establishment of that Chair at Oxford in 1881. His researches on electrical fishes are well known, and, with his brother-in-law, Sir Victor Horsley, he carried out investigations on the physiological connections in the central nervous system. He succeeded to the Waynflete Professorship in 1895, having been Professor of Pnysiology at Liverpool since 1891. As an invaluable teacher and lecturer, and as an enthusiastic member of the various business boards of the University, he did much good work for Oxford. He served on the Departmental Committee of the Board of Trade on Tests for Vision in the Mercantile Marine. His interests outside his special subject were various and many-sided, business and artistic pursuits being equally attractive to him.

### THE FATE OF MORPETH COTTAGE HOSPITAL.

By a narrow majority the governors of Morpeth Cottage Hospital have decided to carry on the institution. At a special meeting last week its position was considered, and a report which had been drawn up on the state of affairs stated that the hospital was a special benefit in the treatment of cases to which the Royal Infirmary, Newcastle, could not attend. On its present lines the committee did not think that it could be carried on, but were willing that a new committee should essay the task which had been too much for them. The principal difficulty has arisen over the admission of patients, and Canon Davies, who presided, remarked that the doctors in the town had not co-operated. Mr. B. C. Oliver's motion that the tenancy of the hospital premises should be terminated was defeated by Dr. Philip's amendment that the hospital be carried on by a new committee. Something very wrong must be the case with the management of a cottage hospital in these days to excite the jealousy or disregard of local medical men, and our experience of such differences has been that a conference between the hospital authorities and the hostile practitioners should be brought about and the matter be readjusted by discussion. The cottage hospital movement has been one of the best friends of the medical profession, and differences between the doctors and the institution must surely be as capable of a settlement at Morpeth as anywhere else.

### THE EVOLUTION OF THE DISPENSARY.

Birmingham has always been a city in which the importance of linking up the various health agencies and after-care organisations has been recognised. The latest addition to its preventive work, since after-care must be included in that term, is the new tuberculosis centre and city analyst's laboratory. It consists of a waiting hall, with consulting, inoculation, and dressing rooms for both sexes and a joint dispensary on the ground floor. The upper part of the building is occupied by Mr. J. Liversege's laboratory as city analyst. The aim, of course, of the centre is preventive and consultative work. It will be a centre for the examination of "contacts" and a general information bureau open to every citizen. As a centre for educational propaganda also it is hoped to do much good work, and during the winter there is a project for holding a course of lectures to patients and their friends. At the present time—for part of the institution has been in use for some six months—nine hundred patients a week are being dealt with. Few things have been more remarkable in institutional development of late years than the way in which the dispensary has grown from a single department into a unit embracing educational, consultative, and specialised branches. The dispensing is now only one part of what is virtually a new institution which the word "centre" not inaptly describes.

### TREASURY GRANTS FOR MEDICAL INSPECTION.

As the result of a letter received by the Surrey Education Committee from the Board of Education, stating that the rate of grant for auxiliary work would not be the same as for medical treatment proper, the Surrey Committee waited upon Sir George Newman, the Board's Chief Medical Officer, to explain the Committee's strong exception to this ruling. Mr. H. A. Powell, the chairman, was able to report to the Education Committee last week that he thought Sir George Newman had been convinced by the deputation that the suggested distinction was impossible. He added an indication that the entire revision of the Treasury's attitude towards grants for medical inspection was a possibility, as he hoped that grants would be made eventually for a comprehensive scheme of medical inspection plus medical treatment. How unsatisfactory—absurd, indeed—the one is without the other it hardly needed the emphasis of experience to prove.

### THE TRAINED NURSE IN THE UNITED STATES.

It may be encouraging to those at present responsible for the efficient training of nurses in this country, especially to the heads of our nurse-training schools, to have their attention called to an extract from a book entitled "The Modern Hospital," by Dr. J. A. Hornsby, the superintendent of the Michael Reese Hospital, Chicago, which we review on another page. This book gives forcible expression to the general dissatisfaction amongst all classes of the community in the United States who employ nurses with the modern-trained nurse as she exists in America to-day. Dr. Hornsby attempts to diagnose the reasons which underlie this general dissatisfaction, which appears to be due in no small degree to the hurried introduction of varying systems of nurse registration under conditions which have not proved in practice to be satisfactory to the nurses or helpful to their employers. Whatever view they may hold as to the principle of nurse registration all who are interested in this question might profitably consider the lamentable state of nursing affairs in the United States at the present time, which is forcibly dealt with in this book.

### AERIAL AMBULANCE.

A new stage in what is called so rhetorically the conquest of the air is being attained by the adaptation of airships or airplanes to passenger traffic and ambulance service. The former we are soon to have an opportunity of judging. The latter, apparently, was tested last week at Aldershot. Of course, the attempt at present is little more than to attach first-aid appliances to the equipment of a biplane, but the doctrine of descent with modification is true of every department of life, and modification has begun, we believe, in the instruments which Colonel J. F. Donegan, R.A.M.C., has designed. A field operating table was an interesting example, compactness being the new aim embodied.

### THE INSPECTOR'S REPORT ON BARNET INFIRMARY.

Miss Wamsley's report, as inspector of the Local Government Board, on the allegations of mismanagement against the Barnet Infirmary, was discussed by the Barnet Guardians last week. The result appears to be that the fibre mattresses are to be replaced by hair; overcrowding has been admitted, and the inmate who does the cooking is to be paid a small sum and to have assistance provided for her. A sub-committee has been appointed to inquire into the long hours of the nursing staff. At another institution upon which Miss Wamsley has been reporting, the Guardians' reply was to the general effect that a new infirmary would be built before long, and that in the meantime matters might be left at a standstill. To be contented with half-hearted and minor reforms is not a worthy alternative to supineness, and we hope that the Barnet Guardians will take steps to put their institution beyond the reach of damaging criticism in the future.

### HOSPITALS IN MINING DISTRICTS.

The growing hospital requirements in mining and colliery districts are presenting problems which are outrunning the attempts to solve them. We may take it as recognised that it is not sufficient to enlarge the existing hospitals of these areas, even though extension may be desirable as part of a progressive scheme. The question then arises as to how the central institutions may be relieved of the strain upon them, and what is at least of equal importance, how the patients may be freed from the unnecessary and sometimes fatal strain of a relatively long journey after a severe accident. The two main suggestions are, of course, for each colliery to have its own cottage hospital, or for what may be described as emergency hospitals to be built in mining areas. The first suggestion has to meet the charge of expense, a charge that has a double edge, from the fact that not merely will the new institution have to be maintained, but the cost of maintaining it may divert funds from the larger hospitals. The suggestion for emergency hospitals was discussed in The Hospital of June 22, 1912, and a plan was published of a small hospital ward, designed to be used as a means to combat shock, the cost of which was estimated at £200. It was further suggested that the provision of such wards should be made compulsory in any works or pithead buildings, since without it first aid must be largely a farce, the risk of non-recovery be greatly augmented, and consequently premiums to insurance companies for compensation needlessly high. It is a pity that Sir Charles Nicholson, when addressing a hospital demonstration at Conisborough the other day, did not enlarge on these points, which lie at the root of the whole problem.

### THE HOSPITAL NEEDS OF HYDE PARK CORNER

At the weekly meeting of the London County Council, which was held on Tuesday, Mr. H. L. Jephson asked Major Ernest Gray, as chairman of the General Purposes Committee, whether the proposed removal of St. George's Hospital would not denude a large area of West and Central London of hospital accommodation at a point where the dangers of street traffic are specially great. He further suggested that the changed condition of affairs called for the Council to reconsider its dilatory attitude in regard to providing an efficient ambulance service. Major Gray replied that a station for emergency cases would, he thought, be preserved in the district, but he held out no prospect of the Council's reconsidering its attitude on the ambulance question. Parliament, he added, was making satisfactory progress with the Council's proposals, and he had every hope that they might be able to see London covered with an efficient ambulance service before the end of the year.

### MUSIC AND THE HOSPITALS.

We have previously remarked upon the way in which music in the past has been used as a means of attracting support for the hospitals. The Birmingham Festival was the classic instance; and, if we remember right, the Beckett Hospital, Barnsley, also has made good use of local musical talent. An effort is now being made to hold an open-air musical festival on the first Sunday in August at Redcar, to which many in East Cleveland and visitors from Darlington are expected to come. It is curious that such attempts should be so characteristic of the Midland and Northern Counties, and to have found so few imitators in the South of England. It will be interesting to learn to what extent the hospitals benefit from this effort; but surely success is unnecessarily jeopardised by the intention of holding the festival in the open air?

### SALARIES FOR VACCINATION OFFICERS.

The Lambeth Board of Guardians have adopted the principle advocated by the Local Government Board to remunerate their vaccination officers by means of fixed salaries instead of payment by fees. The salaries are based on the average sum taken by the officers in fees for five years previous to 1908, when the Vaccination Act came into force. The Guardians have somewhat reluctantly come to this decision, their chief objection being the removal of the inducement for the officers to secure the highest possible number of vaccinations. The salaries are, therefore, to be revised in the event of the combined number of vaccinations and exemptions in any district falling below a minimum of 80 per cent. of the births registered. The exemption certificates in Lambeth in the year 1905 amounted to 114, but last year they had increased to 1,388.

### A GUARDIAN AND HOSPITAL DISCIPLINE.

At a meeting of the Halifax Board of Guardians last week, the Chairman, the Rev. H. F. Wonnacott, accused a member of the Board, Mr. S. Howarth, of discussing the policy of the Board with the epileptic patients. He further stated that Mr. Howarth had told an attendant that he was being underpaid and overworked. The Chairman added that such conduct was disastrous to the welfare of the patients and to the discipline of the institution. Mr. Howarth said that he visited the institution in response to a message from the patients, who described him as their champion. They complained of the way in which the food was cooked; he replied that he was not on the hospital committee, but would mention the matter to the Chairman. He had not spoken to them about the Board. He added that he was "out for the poor." The Clerk pointed out that any complaint must be dealt with by the respective committees, and that interfering individuals would have to be excluded from the institutions of the Board. Mr. Howarth's motives seem good enough, but, in dealing with such cases, there must be method and propriety, especially with complaints and in remedying what may seem to an individual defects in salaries and blots on administration.

### ISOLATION EXTENSIONS AT TONBRIDGE.

In view of the constant discussion as to the best design for isolation hospitals, a problem which is referred to in the articles which Dr. Franklin Parsons is writing in our columns, additional interest attaches to the practically rebuilt isolation hospital at Tonbridge which was opened in the early part of the present month. Situated in its own grounds, the hospital consists of an administrative block of two storeys, connected by covered ways, with a ward block on the east side and a cubical block on the west. The ward block comprises two wards, each of which is 36 feet long by 24 feet wide, a nurses' duty-room, bedroom, and lavatories.

The cubical block, which is composed of six wards, each of which measures 11½ feet by 10½ feet, is furnished with a verandah on to which the wards open. There is also a laundry block, with ambulance shed and coal store, and a discharging block. The latter has a central bathroom, and a dressing and an undressing room. The cubical block has a nurses' room in the centre, and the beds are divided by glass partitions. The cost is £2,600, including £100 for furniture, or practically twice what the original building cost which was opened in 1880. The building was opened by Dr. Watts, chairman of the Council, and a tribute was paid to the work of Mr. Bradley, the County surveyor.

### HOSPITAL PHARMACISTS AND THE BRITISH PHARMACEUTICAL CONFERENCE.

The British Pharmaceutical Conference, the jubilee of which will be celebrated at the annual meeting in London next week, owes much to hospital pharmacists. Both of its honorary secretaries are engaged in hospital work, Mr. Horace Finnemore being chief pharmacist at Guy's and Mr. R. R. Bennett chief pharmacist at University College Hospital, while the chairman of the local committee, which has made all the arrangements for the celebrations, is Mr. Edmund White, president of the Pharmaceutical Society, who was for many years pharmacist at St. Thomas's. On the other hand, hospital pharmacy owes much to the British Pharmaceutical Conference, which has been the medium through which many of the improved processes of manufacture now practised in the pharmaceutical laboratories of hospitals have been introduced. Possibly the Conference is best known to medical men by the "B.P.C." Formulary, which set up standards for commonly prescribed preparations which were not in the Pharmacopœia, and was for many years in constant use in hospitals. The opening function will be a kind of reunion at the Guildhall on Monday evening next, and the two following days will be devoted to the reading and discussion of papers, some of which will be of direct interest to hospital pharmacists.

### RECAPTURE OF ESCAPED MENTAL PATIENTS.

More than one point of interest attaches to the case arising out of the patient who escaped from Colney Hatch Mental Hospital in February, who was subsequently in Wandsworth Infirmary, and has now been convicted at the Central Criminal Court of the murder of a little girl. The jury found him insane at the time. In answer to several questions by the Judge as to the statutory period of recapture, Dr. Gilfillan, the medical superintendent of Colney Hatch Mental Hospital, stated that cases sometimes occurred in which patients got away and evaded recapture over the statutory period, but he had not himself had experience before of such an escape being followed by the commission of a crime. The jury added a rider that the period during which a man might be recaptured should be lengthened from 14 days, and were thanked for doing so. Escapes are sufficiently rare for such a point to be raised as an exception, but it has its importance, and it would be interesting to hear the views of medical superintendents on the subject. Dr. Gilfillan remarked that the Mental Deficiency Bill would not affect this particular point, but that it would certainly deal with large numbers of persons of the same type as the prisoner.

### A NEW PROFESSOR AT EDINBURGH.

Dr. James Ritchie, superintendent of the Laboratory of the Royal College of Physicians, Edinburgh, has been appointed to the new Chair of Bacteriology which has been instituted under the bequest of Mr. Robert Irvine. As a graduate of Edinburgh, and former Professor of and Reader in Pathology in the University of Oxford, Dr. Ritchie will carry to his new post both honours and experience, a double equipment especially necessary in the first holder of a newly founded Chair. Muir and Ritchie's "Manual of Bacteriology" is, of course, a joint production in which he has taken part, and his articles on Immunity are well known to those who follow the scientific literature of the subject in the professional journals. The Irvine Chair of Bacteriology at Edinburgh has started its practical work well in appointing Dr. Ritchie as first Professor.

### THIS WEEK'S DRUG MARKET.

With the exception of a further advance in the price of citric acid, which has been followed by higher quotations for citrates, there have been few price changes of interest during the week. Potassium citrate, sodium citrate, and iron and ammonium citrate are each one penny per pound dearer. Intending buyers of opium are still holding aloof, in the expectation that prices will shortly be lower; in the meantime makers of morphine have reduced their quotations by sixpence per ounce. There is, of course, a possibility that the continued unsettled political conditions in the Near East may have a hardening influence on the value of opium, but buyers are less anxious about this than about the yield of the new crop. The position of cod-liver oil is practically unchanged, but the price tendency seems to be in an upward direction. Croton oil has been slightly reduced in value. The price of sugar of milk tends lower. There has been no change in the position of quinine. It is not altogether improbable that a reduction in the value of cocaine may take place before long, but this is, of course, uncertain. At the recent public sale of drugs in Mincing Lane the demand was, on the whole, dull, the general tendency being towards lower prices.

### TO OUR READERS.

Contributions are specially invited from any of our readers to these columns. They should deal with topical subjects and news. They must be authenticated for the information of the Editor only. The minimum payment if published is 5s. There is no hard-and-fast rule as to space, but notes of about twenty lines in length are preferred.

## Figures and Tables

**Figure f1:**